# Comparison of Nest Defense Behaviors of Goshawks (*Accipiter gentilis*) from Finland and Montana

**DOI:** 10.3390/ani9030096

**Published:** 2019-03-19

**Authors:** Marilyn Wright, Risto Tornberg, Dustin H. Ranglack, Nate Bickford

**Affiliations:** 1 Department of Biology, Utah State University, Logan, UT 84322, USA; wright.marilyn13@gmail.com; 2 Department of Biology, Oulu University, 90014 Oulu, Finland; ristotorn@gmail.com; 3 Department of Biology, University of Nebraska Kearney, Kearney, NE 68849, USA; ranglackdh@unk.edu

**Keywords:** goshawk, Finland, North America, nest defense, aggression

## Abstract

**Simple Summary:**

Understanding the degree to which human interaction may alter natural animal behavior has become increasingly important in developing effective conservation strategies. We examined two populations of northern goshawks (*Accipiter gentilis*) in Montana and Finland. Goshawks in Finland were not protected until the late 1980s, and prior to this protection were routinely shot, as it was believed that shooting goshawks would keep grouse populations high. In the United States, Goshawk were not shot as a management strategy. Though aggressive nest defense has been characterized throughout North America, goshawks in Finland do not show this same behavior. To quantify aggression, we presented nesting goshawks with an owl decoy, a human mannequin, and a live human and recorded their responses to each of the trial conditions. We used statistics to compare the two populations. Our results suggested that goshawks in Montana exhibit more aggressive nest defense behaviors than those in Finland. While this could be due to some biotic or abiotic factor that we were not able to control for in a study on such a small scale, it is also possible that the results from this study suggest another underlying cause, such as an artificial selection pressure created by shooting goshawks.

**Abstract:**

As human impacts on wildlife have become a topic of increasing interest, studies have focused on issues such as overexploitation and habitat loss. However, little research has examined potential anthropogenic impacts on animal behavior. Understanding the degree to which human interaction may alter natural animal behavior has become increasingly important in developing effective conservation strategies. We examined two populations of northern goshawks (*Accipiter gentilis*) in Montana and Finland. Goshawks in Finland were not protected until the late 1980s, and prior to this protection were routinely shot, as it was believed that shooting goshawks would keep grouse populations high. In the United States, Goshawk were not managed as predator control. Though aggressive nest defense has been characterized throughout North America, goshawks in Finland do not show this same behavior. To quantify aggression, we presented nesting goshawks with an owl decoy, a human mannequin, and a live human and recorded their responses to each of the trial conditions. We evaluated the recordings for time of response, duration of response, whether or not an active stimulus was present to elicit the response (i.e., movement or sound), and the sex of the bird making the response. We used *t*-Test with unequal variance to compare mean number of responses and response duration. Our results suggested that goshawks in Montana exhibit more aggressive nest defense behaviors than those in Finland. While this could be due to some biotic or abiotic factor that we were not able to control for in a study on such a small scale, it is also possible that the results from this study suggest another underlying cause, such as an artificial selection pressure created by shooting goshawks.

## 1. Introduction

Human impacts on wildlife have become a topic of increased interest in the last few decades, but, while many studies focus on direct impacts such as overexploitation and loss of habitat, there are fewer studies focusing on the impacts humans have on animal behaviors [1]. There are many examples of human activities that have altered animal behaviors, and often these changes were unintended. Landfills and garbage dumps are regularly visited by animals that have become conditioned to feeding on human waste as opposed to food available in their natural environment [2,3]. Predators such as coyotes (*Canis latrans*) and mountain lions (*Puma concolor*) inhabit urban areas [4,5]. Migratory animals such as birds and elephants will commonly choose routes and stop-over sites with the least amount of human disturbance [1,6,7], and Killer whales (*Orcinus orca*) and Amur tigers (*Panthera tigris* altaica) significantly reduce time spent foraging or consuming a kill to avoid human contact [1,8,9,10]. While some of these changes may be beneficial in some regards, they may also have the potential to affect the survival and reproduction potential of individuals, ultimately affecting population size [1].

Because of the potential negative impacts, as well as the largely unintended alterations humans have caused in animal behavior, considerations of anthropogenic effects are becoming increasingly important for building conservation strategies [1,11,12,13]. A major goal of wildlife management is to minimize human impact to species and mitigate human-wildlife conflict [14,15], so considerations for how we may be inadvertently changing populations are important.

In Europe, grouse (*Phasianidae* spp.) are a staple food source for northern goshawks (*Accipiter gentilis*). Tornberg et al. [16] demonstrated that the dynamics of grouse guilds are coupled with that of goshawks, though the coupling is largely species-specific. Goshawks in Europe have a long history of persecution in relation to individual management of game species such as grouse, especially in farmland areas. For many years, goshawks and other raptors have been shot because it was believed that eliminating predators like goshawks would allow grouse populations to flourish [17,18]. While wintering juveniles were the most commonly targeted birds, some hunters specialized in the location and extermination of breeding goshawks as well [18]. With an increased effort to change this management practice through the combination of legal protection and change in public opinion, Saurola [19] reported a 50% decline in the lethal management of goshawks between 1960 and 1980 [18]. This estimate was based on the recovery rate of goshawks which had been tagged as nestlings and were reported as killed during their first year of life [19].

Though management of the species has changed significantly, there is a possibility that the practice of lethal measures prior to the legal protection to goshawks may have played a significant role in changing the nesting behavior of goshawks of Finland. In the United States, Goshawk were not shot as a management strategy of predator control. Goshawks in North America exhibit a much different pattern of behaviors near nest sites. In response to approaching predators, female goshawks often use a high-pitched call (ca, ca, ca) at rapid intervals. Calls may proceed or accompany an attack such as swooping down to the intruder to deliver a blow from the feet or raking exposed portions of the intruder’s body with the large hind talons [20,21,22,23]. While Northern Goshawk behavior across North America reflects these patterns, goshawks in Finland do not show these behaviors ([18,24] Bickford and Tornberg unpublished data 2015). When approaching nest sites in Finland, females are uncommonly quiet and reserved [18], Bickford and Tornberg personal communication, 2015). Hunters who specialized in killing breeding goshawks under the previous management paradigm likely did so by locating females who displayed the loudest and most effective defense displays while males were out hunting (Tornberg personal communication 2015). One of the potential outcomes for this type of management is the removal of aggressive traits from the Finnish goshawk population, the end result being inadvertent change in the nesting behavior of Finnish goshawks for more docile females that lack aggressive nest defense.

Comparing goshawks in North America and Finland may provide insight into the complex influence of management strategies on population genetics that ultimately control behavior in a species. The knowledge gained from such a study will provide a more comprehensive understanding of the interrelatedness of these aspects to guide management decisions in the future. This study outlines the basis for further investigation into the potential underlying impacts of lethal management of goshawks by demonstrating the differences between Finnish and North American goshawk nest defense. As treatments we used a mechanical owl, human mannequin, and live human (randomizing the order of treatment at each nest) to simulate a suite of predatory advances at nest sites near Oulu, Finland and the Little Belt Mountains, Montana and recorded the responses to each of these predators from adult birds.

## 2. Study Area

**Figure 1:**
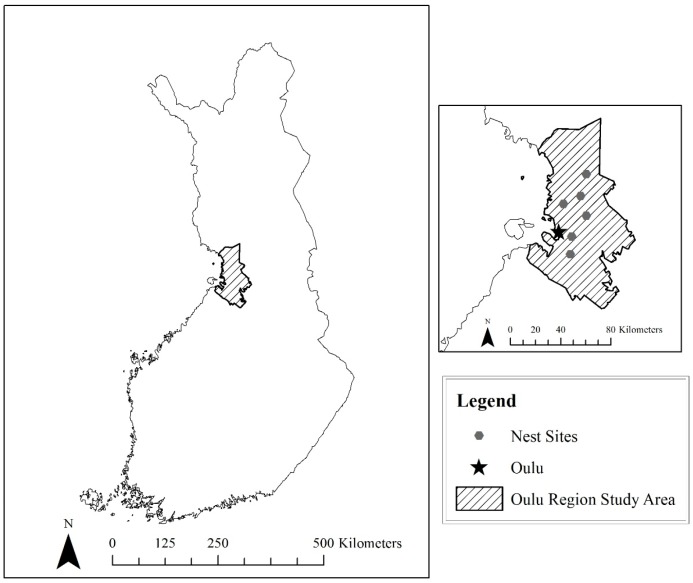
The study area in west-central Finland, near the city of Oulu. Nest sites are approximated locations.

**Figure 2:**
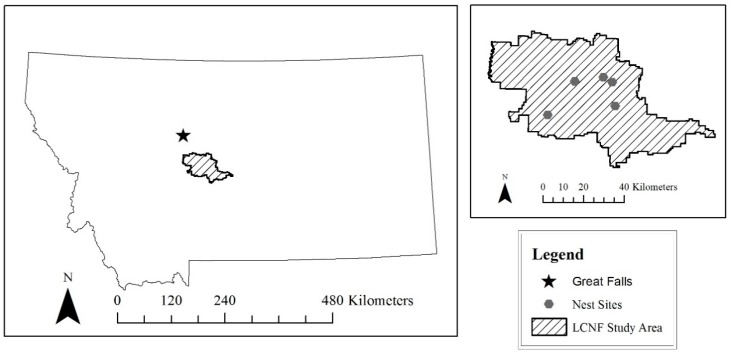
Little Belt Mountains, part of the Jefferson Division of the Lewis and Clark Naitonal Forest, lcoated southeast of Great Falls in central Montana. Nest sites are approximated locations.

We selected two study areas with known nesting Goshawk populations, one near Oulu, Finland and one in the Little Belt Mountains, Montana (Figure 1 and Figure 2). Oulu is located in west-central Finland, and the area around the city of Oulu is heavily forested, dominated by pine (*Pinus silvestris*) and spruce (*Picea abies*). Subject to intensive management that imitates natural forest succession, these stands are composed largely of old-growth timber (Finnish Forestry Association). Six active nest sites were identified, four sites northeast of the city of Oulu and two sites to the southeast.

The Little Belt Mountains are a segment of the Jefferson Division of the Helena-Lewis and Clark National Forest. Located in central Montana southeast of the city of Great Falls, the Little Belt Mountains encompass approximately 40,000 hectares of national forest land and additional privately-owned land. Considered an island range, the Little Belts are a section of the northern Rocky Mountains. Average elevation for the range is around 2500 m with the highest point at 2800 m. The forest is composed primarily of Douglas fir (*Pseudotsuga menziesii*) and lodgepole pine (*Pinua contorta*), but other associated species include white spruce (*Picea glauca*), Engelmann spruce (*Picea engelmannii*), and subalpine fir (*Abies lasiocarpa*) [25]. The US Forest service manges activities in this area including timber harvest, fire suppression, and recreation. Five active nest sites were identified in the Little Belts, two sites near Highway 89 and Monarch and three sites near southwest of Highway 87 in the Judith Basin. No human persecution of goshawks occurred in Montana.

## 3. Methods

To examine the differences in nesting defense behavioral between goshawks in Finland and Montana, three experimental conditions were presented at each occupied nest in the sample areas. All trials were conducted consecutively on the same day with rest intervals of at least 15 min between trials to allow the birds to return to the nest, check on the chicks, and recover from stress. The rest period varied, depending on the degree of response from the birds, with more aggressive responses leading to a longer rest period. Trial periods were twenty minutes long. Time of testing was variable due to 23 h of daylight in Finland during the summer, but testing always occurred during daylight hours (between 6 AM and 5 PM for Montana). Trials took place in Finland from 23–25 May 2016 and from 13 June–6 July 2016 in Montana. We recorded ambient data such as temperature, weather conditions, starting/ending times of each trial, response type, time, and sex of the bird in a field notebook.

The first condition was a natural predator, the Eurasian Eagle Owl (*Bubo bubo*) in Finland and the Great Horned Owl (*Bubo virginianus*) in Montana. These owls are both predators of Goshawk at nest sites [26,27,28,29]. Decoys were prepared from study skins of each species. The skins were stuffed to mimic a live owl, and the basic components from a Lego^®^ remote control set were used to construct a frame to fit the head in order to make it move to the left and right approximately 180°. The owl decoys were set up on a five-foot stick collected from the forest floor at the sampling locations. A predator call box (ICOtec^®^ GC300 Electronic Predator Call, Cabalas, Kearney, NE, USA) with a recorded owl call was placed at the base of the decoy, and calls were played every 2–5 min until there was a response from the goshawk.

The second condition was a human mannequin. A trail camera tripod was used to construct the basic form of a human body. Sweat pants and a hooded sweatshirt were purchased from a local thrift store to give the form a human appearance and shape. A stick (approximately two feet in length) was collected from each sample site to simulate shoulders. The Bescor^®^ MP-1E (Cabelas, Kearney, NE, USA) pan head with extension cable and a foam head (purchased at Hobby Lobby^®^, Kearney, NE, USA) were used to create a more realistic human appearance with left, right, up, and down head movements.

The final condition was a live human. A field technician moved and talked beneath the nest during the trial period. In order to simulate a situation similar to an individual encountering a nest by chance, the volume of the talking, amount and type of movement, and duration of each stimulus varied within and between trials. Although we made an effort to ensure consistency between nest sites and all activities were performed at each nest, the goal was to create a realistic human–nest encounter, so the subject was also permitted to engage in activities such as taking pictures of the adult birds and chicks and collecting leaves and sticks from the forest floor and these were done at all nest sites. The clothes were kept the same for each trial.

Both the owl and human decoys were set up in clear view of the nest (approximately 20–50 feet from the base of the nest tree). The head of the owl and human decoy were moved at random timeframes throughout the study period. A camera was set up directly behind the decoys (approximately 30 feet) at a vantage point that allowed for the clearest view of the decoy and surrounding area. Conditions were presented in a rotating order at subsequent nest sites to prevent order effects due to desensitization. The different treatments may allow us to elucidate natural vs human predation. Observers used camouflage to conceal their presence during trials. In Finland, a soft camouflage blanket and strategic positioning at the base of trees was enough to conceal the observers, while a pop-up ground blind was used in Montana given the difference in terrain.

To quantify aggression, all recordings, both video and direct observations, were evaluated for the following parameters: exact starting time of a given response, type of response, duration of response, whether or not the bird was reacting to a stimulus (i.e., movement of the decoy or sound made by the decoy), and the sex of the bird making the response. Video was used to make sure vocal sounds were not missed during the observation. The starting time of a response was recorded as the minutes and seconds from the start of each experimental trial in which the response began. Types of responses were broken into four broad categories, each with a specific behavioral category. A “kak” was defined as the vocal response characterized as a defense call by Sutton [20] and Schnell [21]. A “high pass” was defined as a pass made at least three meters above the test decoy while a ‘low pass’ was defined as a pass made within three meters of the test decoy without direct contact. A ‘hit’ was defined as direct contact made with a test decoy. Duration of each response was characterized as the start time of each response subtracted from the end time of each response (characterized by at least one second of no interaction). Acting stimuli were recorded as ‘Yes’ if there was some sort of stimulus (movement, sound) produced by the test decoy at least one second prior to a response and ‘No’ if there was no stimulus. The sex of each bird was determined by evaluating the size of the bird as well as the pitch of the ‘kak’ call.

The data were analyzed based on each individual nest site of which there were six in Finland and five in Montana. *t*-Test with unequal variance was used to compare the mean number of responses and response duration of birds at Finnish and Montana nest sites.

## 4. Results

Responses were recorded at six nest sites in Finland and four in Montana. All recorded responses from both the populations in Finland and Montana showed high variation, with some birds having a ‘zero response’ under each experimental condition. The condition with the most active responses from different birds both in Finland and Montana was the natural predator or owl decoys. At three sites in Montana, we recorded passes made at the owl decoy. The majority of passes made were low passes, made by both male and female birds (Table 1). We recorded our most aggressive response of all combined trials and sites at one of our Montana sites. At 13:41 min, the female bird at one of the nest sites in Montana made physical contact with the head of the owl, knocking the decoy off of the post and detaching the head, which was found nearly twenty feet from the decoy, suggesting that she either had a hold of the head for a brief moment or struck it with enough force to displace it. Birds in Montana, both male and female, were recorded as making several high and low passes at decoys at all but one site, while no birds in Finland made passes at any of the decoys (Table 1).

Responses towards the human mannequin showed greater variation, both within and between samples. The greatest total number of responses for the sites in Finland were recorded in response to the human mannequin, but only three of the six total nesting birds surveyed responded during the trials. We recorded responses at three of the nest sites in Montana, including responses from male birds at two sites. At these same two sites, both high and low passes were made at the mannequin (Table 1).

Responses towards the live human demonstrated the most variation between samples. At three of the sites sampled in Finland, only one ‘kak’ response was recorded. In Montana, responses were recorded at three sites, with responses from both the male and female birds at one of the sites. Both high and low passes were made at the site 1 and site 2, with more passes made at the site 1 (Table 1).

The *t*-Test with unequal variance for number of responses between sites in Finland and sites in Montana was significantly different (*p* = 0.024, t-crit = 2.14, df = 14) but the *t*-Test for response duration was not significant (*p* = 0.09, t-crit = 2.04, df = 31).

## 5. Discussion

Throughout this study, the Goshawk population sample in Montana was consistently more aggressive than the population sample studied in Finland. This was evident in comparisons of raw data including number of responses, number of passes, and duration of responses, all of which were greater for the goshawks in Montana. The trial with the owl decoy elicited the most aggressive responses from birds at both sites, but birds in Montana where far more aggressive. It was the only trial in which a bird made physical contact with a decoy. The randomness of owl head movement, mannequin movement, and human movement could have been different between nest sites. However, the basic movement and the numbers of movements was kept similar between nest sites and we do not feel that this could have been the difference between response and nonresponse.

Statistical analysis demonstrated a significant difference in the mean number of responses but not the duration of responses. The difference in the mean number of responses between Finland and Montana, with a higher mean number of responses in Montana, suggests that birds in Montana do react more often and more aggressively towards perceived threats of predation such as those from owls and humans.

There were birds in both the Finnish and North American samples that had zero response to each of the experimental conditions. While this suggests lack of aggression in the Finnish population sample, it may be some other variable affecting the birds from the nest sites in Montana. At one of the Montana nests, both the male and female birds had responded aggressively when the field team initially located the nest, but there was no interaction after that under any of the experimental conditions. As the chicks were still fairly young, there was an expectation of aggression, but it could possibly be related to abnormal weather patterns in the Little Belts including several overcast and rainy days during what is typically a drier season. The other nest site in Montana where a response was not recorded was the last nest visited during the sampling season, and, by the time the nest was located, the chicks were very near fledging. At this stage, the females become less aggressive in nest defense and allocate more time to hunting away from the nest site [21].

The lack of aggressive nest defense in Finnish birds presents an interesting area for future research. One possible reason for the lack of aggressive nest defense is that within the population the aggressive birds were shot and those that are less aggressive birds survived to reproduce. It could also be a learned response within the Finnish Goshawk population. While many aggressive birds have likely been shot in Finland, it is likely that some birds survived being shot at and may have learned to associate aggressive nest defense towards humans with a greater inherent risk. Even though this behavior may not be inherited, the genetics for the ability to learn could be inherited. Also, if the parent birds do not attack humans the young birds will not learn the associated aggressive behavior to humans. This may explain why it appears Finnish goshawks seemed more likely to react to their natural predators, Eurasian Eagle Owls, as opposed to the mannequin and the live human.

There are many examples of studies that have linked specific management decisions to genetic changes within a species. One of the most well-known examples is the case of changing population genetics in bighorn sheep (*Ovis canadensis*). Phenotype-based harvests such as sport trophy hunting of rams selects for desirable traits such as body weight and horn size. A 30-year study of a wild population of bighorn sheep in which trophy hunting targeted rams with large horns demonstrated a decline of mean horn size and body weight in the population over time. Trophy rams were generally considered to be of “higher breeding value” in regard to weight and horn size, and their removal from the population due to unrestricted trophy hunting resulted in lighter weight rams with smaller horns. Over the duration of the study, fewer trophy rams were produced from the population because the heritable traits of large body and horn size were removed from the gene pool [30].

In avian populations, a variety of behaviors have been demonstrated to have a genetic basis including migration and tool use [31,32]. By inherently removing the most aggressive and loudest birds to shoot in Finland, humans have created a pressure on the population which can be described as unintended artificial selection. Artificial selection in animals has been generally understood in the context of domestication, the process by which organisms are removed from the influence of natural selection pressures and subjected to new artificial selection pressures controlled by humans, thus causing evolutionary changes based on this shift in selection pressure [33,34,35,36,37,38]. Several studies have demonstrated that this shift to artificial selection through domestication involves distinct changes in morphology, physiology, and behavior [36,37,39,40,41,42,43,44,45,46]. Despite the studies that have examined artificial selection in a manipulated environment, few studies have considered the possibility that certain management practices imposed on wild animal populations may exert similar artificial selection pressure effects. One of the conditions for evolution is variation within a trait (Stearns 1992). In our study, there was variation in aggressive response, both Finnish and North American Goshawk population samples. This lends support to the idea that aggressive nest defense is heritable and therefore could be lost as the result of selective pressure.

While many of the implications from this study do not inherently suggest a link between the shooting of Finnish goshawks and their deviance from aggressive nest defense, the clear demonstration of less aggression as compared to birds in North America warrants further investigation and calls attention to the potential unintended impacts of certain management decisions. A larger sample size including multiple study locations both in Europe and the United States would provide better insight into the extent of birds lacking aggressive nest defense, but funding for large scale avian behavioral projects tends to be meager at best. It is possible that the differences observed between these two populations had some environmental cause that could not be controlled for or measured at such a small scale. Finland is located at a higher latitude and receives more sunlight than Montana during nesting season. Due to time constraints, we had to sample the Montana sites later in the nesting season than we sampled Finland. This required the use of a call box at sites in Montana to attract attention from goshawks who were able to be away from their chicks longer than the Finnish birds, who had younger chicks at the time of sampling and were always on or near the nest during before our trials began. There are also differences in terrain, elevation, precipitation, and many other biotic and abiotic environmental factors which could possibly account for behavioral differences between the two populations. However, despite the limitations of observation-based behavior studies such as this one, we were still able to provide a foundation for exploring behavioral differences which may have been affected by human impact. Future research should investigate the underlying causes of these differences including, but not limited to, comparison of hormones underlying avian aggression between the two populations such as glucocorticoids and gonadal hormones [47,48,49,50,51,52,53,54]. This would allow for a better understanding of how human interactions may play a role in wildlife population genetics which may be a vital component of conservation and management that is currently understudied.

## Figures and Tables

**Table 1:** Total responses, average (with CI) numbers of responses (“kaks” and passes), and response duration per nest by females and males at nest sites in Finland (*n* = 6) and Montana (*n* = 5).

Treatment Type	Owl		Mannequin		Human	
Location	Finland	Montana	Finland	Montana	Finland	Montana
Total Responses	9	118	12	122	1	180
Average Number of “kaks”	1.5 ± 1.8	19.2 ± 13.5	2 ± 1.9	19.6 ± 12.2	0.17 ± 0.4	33.4 ± 24.2
High passes	0	0.8 ± 1.2	0	1.6 ± 1.2	0	1 ± 0.9
Low passes	0	3.2 ± 5.2	0	1.2 ± 2.1	0	1.6 ± 1.5
Average Response Duration by males	0	6.9 ± 2.9	0	6.8 ± 3.1	0	3 ± 1.4
Average Response Duration by females	5.3 ± 1.9	4.3 ± 2.1	4.8 ± 0.9	7.6 ± 2.7	1	5.3 ± 2.9
